# Supplementary material for: Impact of Grip Strength in Patients with Unresectable Hepatocellular Carcinoma Treated with Lenvatinib
Source: Cancers (Basel). 2020 Aug 3;12(8):2146. doi: 10.3390/cancers12082146 (PMC7465794; doi:10.3390/cancers12082146)
Supplement: Supplementary file 1 [file cancers-12-02146-s001.pdf]

cancers

Supplementary Materials

Impact of Grip Strength in Patients with Unresectable Hepatocellular Carcinoma Treated with Lenvatinib

Table S1. Adverse effects observed in more than 15% of the all patients.

	**All Grade**	**Grade 3/4**
Hypertension	22 (34.9%)	5 (7.9%)
Thyroid function abnormality	19 (30.2%)	1 (1.6%)
Fatigue	18 (28.6%)	1 (1.6%)
Urine protein	14 (22.2%)	6 (9.5%)
Diarrhea	12 (19.0%)	1 (1.6%)
HFSR	11 (17.5%)	2 (3.2%)
Appetite loss	11 (17.5%)	2 (3.2%)

HFSR, Hand-foot skin reaction.

© 2020 by the authors. Licensee MDPI, Basel, Switzerland. This article is an open access article distributed under the terms and conditions of the Creative Commons Attribution (CC BY) license (http://creativecommons.org/licenses/by/4.0/).
